# DNA Barcoding and Comparative Chloroplast Marker Performance in Endemic Plants of Crete (Greece)

**DOI:** 10.3390/cimb48050500

**Published:** 2026-05-13

**Authors:** Dimitra Ioannidou, Ioulietta Samartza, Georgios Tsoktouridis, Andreas D. Drouzas, Nikos Krigas

**Affiliations:** 1 Laboratory of Systematic Botany and Phytogeography, Department of Botany, School of Biology, Aristotle University of Thessaloniki, GR-54124 Thessaloniki, Greece; demy.ioann@gmail.com; 2 Institute of Plant Breeding and Genetic Resources, Hellenic Agricultural Organization Dimitra (ELGO-Dimitra), GR-57001 Thessaloniki, Greece; isamartza@elgo.gr (I.S.); gtsok@elgo.gr (G.T.); 3 Institute of Olive Tree, Subtropical Crops and Viticulture, Hellenic Agricultural Organization Dimitra (ELGO-Dimitra), GR-71307 Heraklion, Greece; 4 Department of Agriculture, School of Agricultural Sciences, Hellenic Mediterranean University, GR-71410 Heraklion, Greece

**Keywords:** cpDNA, Lamiaceae, Campanulaceae, biodiversity, phylogeny

## Abstract

Crete, a major Mediterranean biodiversity hotspot, hosts many local endemic, threatened and/or protected plant taxa (species and subspecies). Besides their ecological and conservation significance, these unique phytogenetic resources hold significant economic potential for sustainable utilization. Since DNA barcoding is critical for conservation, taxonomy, and plant-derived product authentication, we studied 15 local Cretan endemic taxa using three chloroplast DNA (cpDNA) regions (*rbc*L, *trn*L, *trn*H-*psb*A). A comparative analysis against GenBank (NCBI) records revealed significant new data: (i) the first genetic information for five taxa (*Centaurea redempta* subsp. *redempta*, *Galium fruticosum*, *Micromeria hispida*, *Salix kaptarae*, *Teucrium cuneifolium*); (ii) new marker-specific sequences for seven taxa (*Helichrysum heldreichii*, *Scutellaria hirta*, *Sesleria doerfleri*, *Staehelina petiolata*, *Teucrium alpestre*, *Campanula pelviformis*, *Phlomis lanata*); and (iii) novel genotypes of already represented markers for three species (*Phlomis lanata*, *Scutellaria sieberi*, *Staehelina petiolata*). Phylogenetic analyses were performed for all three molecular markers across selected members of *Scutellaria* section *Scutellaria*, *Teucrium* section *Polium*, and *Campanula* section *Quinqueloculares.* The overall results indicated that, amongst the studied species, the *trn*H-*psb*A marker is more suitable for species-level identification, whereas the *rbc*L and *trn*L markers were more helpful to genus-level identification within Lamiaceae and Campanulaceae. These results enrich the DNA barcoding reference library and form a concrete contribution towards the protection, conservation and traceability of Crete’s unique botanical heritage.

## 1. Introduction

Crete, the largest Greek island and the fifth largest in the Mediterranean region, has been recognized by the IUCN (International Union for the Conservation of Nature) as a Global Centre of Plant Diversity and a major Mediterranean biodiversity hotspot [1], hosting at least 227 endemic taxa (species and subspecies) to date [2,3,4,5]. This remarkable level of plant endemism is driven by the diverse geomorphology, intense tectonic activity and long-term geographical isolation of the island [2,6], which together have generated a wide range of habitats and ecological niches. However, several populations of these local endemic plants face multiple anthropogenic threats, including overexploitation [7,8] and intense tourism pressure, challenges that are common among Mediterranean islands [2,9]. Conservation-wise, these factors are widely recognized as primary drivers of biodiversity loss at global and local scales [1].

From a conservation perspective, the taxonomic delimitation of local endemic plant taxa constitutes a fundamental process in scientific research across various disciplines, while accurate and reliable identification of plant species is fundamental, representing the basis for correct interpretation of scientific results. Since 2003, DNA barcoding has been introduced as a new method for species identification at molecular level using short, standardized DNA sequences as unique “barcodes” to distinguish among organisms [10].

In plants, no single universal barcode equivalent to the animal mitochondrial COI region has been established to date; thus, the focus has been on the chloroplast genome (cpDNA), and a number of regions have been suggested as barcoding regions (e.g., *rbc*L, *trn*L, *trn*H-*psb*A, *mat*K, etc.). The properties of the cpDNA markers (uniparental inheritance, small size, relatively low mutation rate, abundance in the plants) renders them ideal for DNA barcoding. When, however, results started accumulating, it turned out that the effectiveness of DNA barcoding in plants is dependent on the region used as well as on the studied taxa [10,11,12]. Thus, none of the proposed cpDNA regions (either coding or non-coding) alone proved to be efficient for species-level identification, and, subsequently, the combined use of more than one cpDNA region and of the nuclear genome (nDNA) ITS region has been proposed to improve discriminatory power [10,11,12]. But even this approach was not efficient enough to determine a particular combination of regions able to identify the majority of plant taxa [11].

The chloroplast genes *rbc*L and *trn*L and the intergenic spacer *trn*H-*psb*A are three of the widely used DNA barcodes for plant species identification (e.g., [10,11]), with ample amounts of information deposited in the GenBank. DNA barcoding overcomes limitations of traditional morphological identification by enabling rapid and accurate species determination through the analysis of specific DNA markers [12,13,14]. This approach has additional applications, such as the identification of differences among related taxa, the detection of cryptic species, the identification of plant species in environmental samples, the construction of phylogenetic trees, and the contribution to biodiversity conservation [14,15,16]. In plants, DNA barcoding is particularly valuable for reliable identification when parts of plant materials (e.g., roots, seeds, pollen) are available and is applied in verifying the authenticity of plant-based or plant-derived products, thus preventing consumer deception and/or illegal trade of threatened plant taxa [17,18].

Despite the value of the local endemic plants and the importance of the Cretan biodiversity hotspot in the contexts of the Mediterranean region, Europe or globally, the studies applying DNA barcoding to or aiming at identifying endemic taxa of the Cretan flora remain surprisingly limited [19,20,21]. Consequently, comprehensive molecular reference data for Cretan single-island endemic plants are still fragmentary, with several taxa lacking any accession in DNA sequence repositories found online.

In this context, the present study aimed to address this gap by generating and analyzing plastid DNA barcode data for 15 local endemic taxa of Crete, using three cpDNA markers (*rbc*L, *trn*L, and *trn*H-*psb*A) which are widely used as DNA barcodes (e.g., [10,11]) and are represented by an ample amount of information deposited in the GenBank. These taxa were specifically selected because they hold promising potential for sustainable utilization in different economic sectors and either lacked prior molecular records or were underrepresented in publicly available databases, thus maximizing the contribution of this study to reference-library development. Beyond data generation, this study aimed at evaluating the effectiveness of these chloroplast markers in discriminating among closely related endemic taxa belonging to taxonomically challenging groups. These include species within *Campanula* L. sect. *Quinqueloculares*, members of *Scutellaria* L. sect. *Scutellaria*, and taxa of *Teucrium* L. sect. *Polium*, all representing cases where morphological identification can be problematic. The inclusion of both taxonomically complex groups and more distinct lineages, such as the monotypic *Petromarula pinnata* (L.) A. DC., allows for a more comprehensive assessment of barcode performance across different evolutionary contexts. Overall, the primary scope of this study was to generate and deposit novel plastid barcode sequences for a set of poorly studied Cretan endemic taxa, thereby enhancing knowledge and molecular reference resources for these taxa. Such data may provide a necessary foundation for follow-up studies and activities in systematics, conservation genetics and management, and biodiversity monitoring where accurate species identification is critical.

## 2. Materials and Methods

### 2.1. Selection of Taxa and Collection of Living Plant Material

The selection of the local endemic taxa for this study was based on pre-defined criteria (Table 1, Table 2 and Table 3) to ensure that their study would be of both scientific and applied significance. To this end, we considered or reviewed for each of these taxa: (i) the availability of information on the reproductive biology and/or pilot propagation and ex situ cultivation (Table 1), (ii) the species-specific extinction risk status (Table 1), (iii) the potential economic value in different industrial sectors and at different levels (Table 2), and (iv) the currently available genetic sequences deposited in GenBank (Table 3).

**Figure 1:**
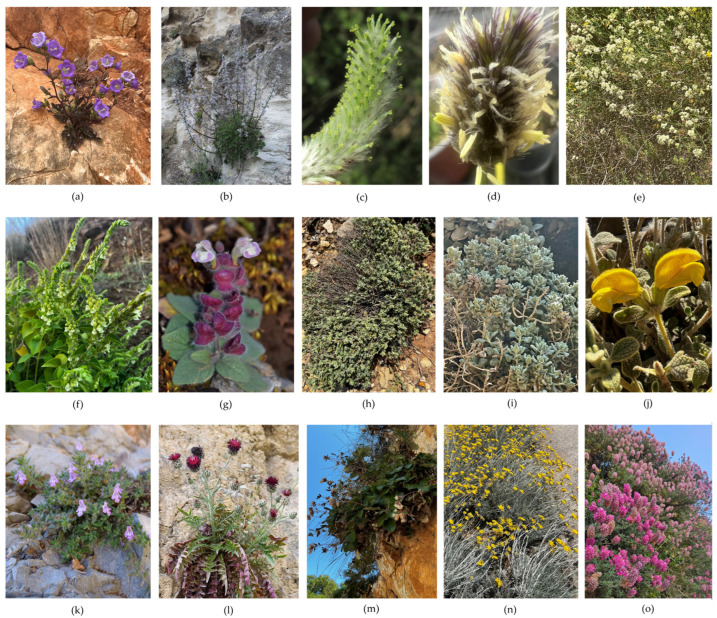
Indicative photographs of the investigated 15 local endemic plants of Crete in their wild habitats. (**a**): *Campanula pelviformis*, (**b**): *Petromarula pinnata* (Campanulaceae), (**c**): *Salix kaptarae* (Salicaceae), (**d**): *Sesleria doerfleri* (Poaceae), (**e**): *Galium fruticosum* (Rubiaceae), (**f**): *Scutellaria hirta*, (**g**): *Scutellaria sieberi*, (**h**): *Teucrium alpestre*, (**i**): *Teucrium cuneifolium*, (**j**): *Phlomis lanata*, (**k**): *Micromeria hispida* (Lamiaceae), (**l**): *Centaurea redempta* subsp. *redempta*, (**m**): *Staehelina petiolata*, (**n**): *Helichrysum heldreichii* (Asteraceae), (**o**): *Ebenus cretica* (Fabaceae).

A total of 15 local endemic Cretan plants of interest were collected from different parts of the island (Figure 1) and were taxonomically identified using standard flora [22]. The collection of living wild-growing plant material was made using special collection permits, which are issued annually by the competent Greek authorities (e.g., YPEN/DPD/182336/879 of 16 May 2019; YPEN/DPD/64886/2959 of 6 July 2020; YPEN/DPD/26895/1527 of 21 April 2021; YPEN/DPD/15539/845 of 24 February 2022; YPEN/DPD/38262/2306 of 2 August 2023; YPEN/DPD/80381/5557 of 9 August 2024; YPEN/DPD 73029/4891 of 1 July 2025).

These living plant materials were propagated and were ex situ maintained with IPEN (International Plant Exchange Network) accession numbers [3] including both living plant material in the conservation unit at the premises of the Institute of Plant Breeding and Genetic Resources of the Hellenic Agricultural Organization Dimitra (ELGO-Dimitra) in Thessaloniki, Greece, and voucher specimens deposited in the institute’s herbarium (acronym: BBGK) (Table 1, Table 2 and Table 3, Figure 1). One individual of each taxon was sequenced; thus, all the data represent single accessions.

**Table 1:** Overview of information regarding plant propagation (SID: Seed Information Database, https://ser-insr.org/seed-information-database, accessed on 6 March 2026), pilot ex situ cultivation and extinction risk according to ecological models [23] and/or the global IUCN (International Union for the Conservation of Nature) Red List criteria (https://www.iucnredlist.org/, accessed on 5 March 2026) defining the extinction risk status (LC: Least Concern; NT: Near Threatened; VU: Vulnerable; EN: Endangered; CR: Critically Endangered) regarding the 15 studied Cretan local endemic plants (in alphabetical order based on their scientific names).

Taxon	Cretan Range	IPEN Number	Plant Propagation and Pilot Ex Situ Cultivation	Extinction Risk According to	
				Ecological Models [23]	IUCN Red List
*Campanula pelviformis* Lam.	Scattered across the island	GR-1-IPBGR(BBGK)- 22,70	[24]	VU	LC
*Centaurea redempta* Heldr. subsp. *redempta*	Western Crete	GR-1-IPBGR(BBGK)- 19,1184	Present study	EN	LC
*Ebenus cretica* L.	Scattered across the island	GR-1-IPBGR(BBGK)- 19,1149	[25]	VU	LC
*Galium fruticosum* Willd.	Scattered across the island	GR-1-IPBGR(BBGK)- 19,109	SID, Present study	EN	-
*Helichrysum heldreichii* Boiss.	Eastern Crete	GR-1-IPBGR(BBGK)- 23,160	Present study	EN	-
*Micromeria hispida* Boiss. & Heldr. ex Benth.	Central-Eastern Crete	GR-1-IPBGR(BBGK)- 23,28	Present study	CR	NT
*Petromarula pinnata* (L.) A. DC.	Scattered across the island	GR-1-IPBGR(BBGK)- 19,124	[26]	VU	LC
*Phlomis lanata* Willd.	Scattered across the island	GR-1-IPBGR(BBGK) 23,102	SID, Present study	VU	LC
*Salix kaptarae* Cambria, C. Brullo & Brullo	Western Crete	GR-1-IPBGR(BBGK)- 21,74	Present study	CR	CR
*Scutellaria hirta* Sm.	Scattered across the island	GR-1-IPBGR(BBGK)- 23,155	[27]	CR	LC
*Scutellaria sieberi* Benth.	Scattered across the island	GR-1-IPBGR(BBGK)- 23,154	Present study	VU	LC
*Sesleria doerfleri* Hayek	Western Crete	GR-1-IPBGR(BBGK)- 19,353	[28]	EN	-
*Staehelina petiolata* (L.) Hilliard & B. L. Burtt	Scattered across the island	GR-1-IPBGR(BBGK)- 19,88	[29,30]	EN	LC
*Teucrium alpestre* Sm.	Scattered across the island	GR-1-IPBGR(BBGK)- 20,382	Present study	VU	LC
*Teucrium cuneifolium* Sm.	South-Western Crete	GR-1-BB IPBGR(BBGK)- 19,1157	[28]	VU	LC

**Table 2:** Overview of the three-level multifaceted evaluations [31] for the 15 Cretan local endemic plants investigated in the present study (in alphabetic order according to their scientific names) outlining their potential (Level I evaluations expressed as relative percentage compared to ideal maximum scoring) in the medical–cosmetic [32], agro-alimentary sector [33], and ornamental–horticultural sector [31], with indications regarding the current feasibility (Level II evaluations expressed as percentage compared to ideal conditions) and estimated readiness timescale for species-specific value chain creation (Level III evaluations); *Salix kaptarae* has not been assessed to date [3].

	General Potential in Different Sectors of Economy—Level I Evaluations (%)			Value Chain Feasibility—Level II Evaluations (%)	Readiness for Value Chain Creation—Level III Evaluations
Taxon	Medicinal–Cosmetic	Agro-Alimentary	Ornamental–Horticultural	Cross-Sectorial	Cross-Sectorial
*Campanula pelviformis*	38.89	42.86	41.67	43.06	Long term
*Centaurea redempta* subsp. *redempta*	16.67	14.29	37.50	33.33	Indeterminate
*Ebenus cretica*	27.78	14.29	60.83	45.83	Long term
*Galium fruticosum*	16.67	23.81	35.83	44.44	Long term
*Helichrysum heldreichii*	38.89	52	36.67	61.11	Short term
*Micromeria hispida*	27.78	54.76	30.00	45.83	Long term
*Petromarula pinnata*	37.04	42.86	52.50	45.83	Long term
*Phlomis lanata*	46.30	35.71	51.67	51.39	Middle term
*Scutellaria hirta*	5.56	30.95	33.33	29.17	Indeterminate
*Scutellaria sieberi*	29.63	30.95	32.50	36.11	Long term
*Sesleria doerfleri*	7.41	0	47.50	56.94	Short term
*Staehelina petiolata*	22.22	14.29	51.67	54.17	Middle term
*Teucrium alpestre*	40.74	42.86	40.00	36.11	Long term
*Teucrium cuneifolium*	40.74	50.00	26.67	63.89	Short term

**Table 3:** Availability (absolute number) of DNA sequences submitted to GenBank for the 15 local endemic species and subspecies of Crete for three barcoding markers (*trn*L, *trn*H-*psb*A, *rbc*L) in NCBI (https://www.ncbi.nlm.nih.gov, accessed on 1 March 2025).

Family	Taxon	*trn*L	*trn*H-*psb*A	*rbc*L
Campanulaceae	*Campanula pelviformis*	1	0	1
Asteraceae	*Centaurea redempta* subsp. *redempta*	0	0	0
Fabaceae	*Ebenus cretica*	2	1	1
Rubiaceae	*Galium fruticosum*	0	0	0
Asteraceae	*Helichrysum heldreichii*	0	0	0
Lamiaceae	*Micromeria hispida*	0	0	0
Campanulaceae	*Petromarula pinnata*	4	1	5
Lamiaceae	*Phlomis lanata*	1	5	0
Salicaceae	*Salix kaptarae*	0	0	0
Lamiaceae	*Scutellaria hirta*	2	0	0
Lamiaceae	*Scutellaria sieberi*	5	3	3
Poaceae	*Sesleria doerfleri*	0	0	0
Asteraceae	*Staehelina petiolata*	0	1	0
Lamiaceae	*Teucrium alpestre*	2	0	0
Lamiaceae	*Teucrium cuneifolium*	0	0	0

### 2.2. DNA Extraction

Young leaves were sampled per taxon from the ex situ-maintained mother plants of wild origin and were taken to the laboratory for grinding with a mortar and pestle using liquid nitrogen. DNA extraction was carried out using the Doyle and Doyle protocol (1987) with minor modifications [34], namely one (1) h incubation in CTAB buffer (AppliChem GmbH, Ottoweg 4, D-64291 Darmstadt, Germany), treatment with Proteinase K (AppliChem GmbH, Ottoweg 4, D-64291 Darmstadt, Germany) and two chloroform–isoamyl alcohol extractions (PanReac Química SLU C/Garraf 2, Polígono Pla de la Bruguera, E-08211, Barcelona, Spain) to effectively remove the proteins. The success of the extraction was assessed through electrophoresis on 0.8% agarose gel in 1× TBE (AppliChem GmbH, Ottoweg 4, D-64291 Darmstadt, Germany) with ethidium bromide (EtBr) (AppliChem GmbH, Ottoweg 4, D-64291 Darmstadt, Germany) running at 70 V/cm. The quantity and the quality (ratio A260/A280) of the extracted DNA were also assessed using Nanodrop 2000c (Thermo Fisher Scientific, Inc., Waltham, MA, USA).

### 2.3. DNA Barcodes and PCR

Since the primary aim of this study was to provide plastid DNA barcoding data, three DNA barcodes were used for DNA barcoding of the 15 local endemic taxa of Crete, the chloroplast genes *trn*L and *rbc*L and the intergenic spacer *trn*H-*psb*A. Preliminary experiments using the *mat*K region of cpDNA gave poor amplification results, and thus, this region was excluded. Amplification of the *trn*L region was conducted using 10 ng of the template DNA, 2.5 mM MgCl_2_ (Kappa Biosystems, Wilmington, MA, USA), 0.4 μM of each primer (*trn*Lc: CGAAATCGGTAGACGCTACG; *trn*Ld: GGGGATAGAGGGACTTGAAC), 0.2 mM of dNTPs (Enzyquest P.C., STEPC, N.Plastira 100, GR-70013, Heraklion, Greece) and 1U Taq polymerase (Kappa Biosystems, Wilmington, MA, USA) in a total volume of 25 μL. For the amplification of the *rbc*L region, 20 ng of the template DNA was used with 2 mM MgCl_2_ (Kappa Biosystems, Wilmington, MA, USA), 0.2 μM of each primer (*rbc*L1F: TGTCACCACAAACAGAAAC, *rbc*L724R: TCGCATGTACCTGCAGTAGC), 0.2 mM of dNTPs (Enzyquest P.C., STEPC, N.Plastira 100, GR-70013, Heraklion, Greece), and 1U Taq polymerase (Kappa Biosystems, Wilmington, MA, USA) in a total volume of 25 μL. Finally, the amplification of the *trn*H-*psb*A region was conducted using 25 ng of the template DNA, 2 mM MgCl_2_ (Kappa Biosystems, Wilmington, MA, USA), 0.1 μM of each primer (*psb*A: GTTATGCATGAACGTAATGCTC, *trn*H2R: CGCGCATGGTGGATTCACAATCC), 0.2 mM of dNTPs (Enzyquest P.C., STEPC, N.Plastira 100, GR-70013, Heraklion, Greece) and 1U of Taq polymerase (Kappa Biosystems, Wilmington, MA, USA) in a total volume of 25 μL.

The amplification protocol for the *trn*L region encompassed an initial denaturation at 94 °C for 2 min, followed by 30 cycles of denaturation at 92 °C for 30 s, annealing at 55 °C for 30 s, extension at 72 °C for 2 min, and a final extension at 72 °C for 10 min [35]. The amplification protocol for the *rbc*L region included an initial denaturation at 94 °C for 5 min, followed by 30 cycles of denaturation at 94 °C for 1 min, annealing at 54 °C for 1 min, extension at 72 °C for 1 min and 30 s, and a final extension at 72 °C for 7 min [36]. Finally, the amplification protocol for *trn*H-*psb*A region involved an initial denaturation at 94 °C for 5 min, followed by 40 cycles of denaturation at 94 °C for 30 s, annealing at 50 °C for 30 s, extension at 72 °C for 30 s, and a final extension at 72 °C for 7 min [36]. None of the above temperatures were changed/optimized from the standard protocols.

The amplification success was examined through electrophoresis in 2% agarose gel with 1× TBE solution (AppliChem GmbH, Ottoweg 4, D-64291 Darmstadt, Germany) and 0.5 ng/μL ethidium bromide (EtBr) (AppliChem GmbH, Ottoweg 4, D-64291 Darmstadt, Germany) for 30 min in 70 V/cm. The PCR products were cleaned up with the use of the NucleoSpin Gel and PCR clean-up kit (Macherey-Nagel, Düren, Germany).

### 2.4. Sequencing

The PCR products of the 15 local Cretan endemic plants for all three DNA barcodes were Sanger-sequenced in both forward and reverse directions by an external provider (Eurofins Genomics, Ebersberg, Germany).

The sequencing results were checked, manually corrected (when needed), and aligned using the BioEdit (v7.7.1) software [37]. The sequences were aligned using the ClustalW algorithm (as implemented in BioEdit), and, before the phylogenetic analyses, the sequences were trimmed to the point where all taxa had the same nucleotides. Indels were retained as gap characters in the alignment.

### 2.5. Data Analysis

The taxonomic identity of each sequence of the respective Cretan endemic taxa was checked using the BLASTn online tool from the National Centre for Biotechnology Information (NCBI), considering both query coverage and sequence identity.

The available sequences from the GenBank database were retrieved, compared and combined with the relevant data produced in the present work (Table 3) with the aim to reveal new contributions in genetic terms, to check for possible differentiation in closely related taxa of some genera (*Campanula*, *Scutellaria*, *Teucrium*), and to carry out phylogenetic analyses.

To check for evolutionary relationships among the 15 local endemic Cretan taxa, phylogenetic analyses were conducted for the two barcode regions *trn*L and *rbc*L (individually and in combination). In addition, comparative phylogenetic trees were constructed for selected taxa, incorporating sequences of closely related taxa from the same section and genus retrieved from the GenBank. Specifically, for the phylogenetic tree of *C. pelviformis*, additional taxa with available genetic data belonging to the section *Quinqueloculares* were included [38]. For the cases of *T. alpestre* and *T. cuneifolium*, additional taxa from the section *Polium* were used [39], and for *Scutellaria hirta* and *S. sieberi*, additional taxa with available genetic data belonging to the section *Scutellaria* were incorporated [40]. All the above analyses were conducted using MEGA (v11) software [41]. All the analyses were performed in MEGA [41]. For each dataset, the nucleotide substitution model with the lowest Bayesian Information Criterion (BIC) score was selected. The K2 + G (Kimura 2-parameter with gamma distribution) model was used for *rbc*L. The HKY + G + I (Hasegawa–Kishino–Yano with gamma distribution and invariant sites) model was applied to *trn*L and to the combined *trn*L + *rbc*L dataset. The HKY + I (Hasegawa–Kishino–Yano with invariant sites) model was used for the combined *trn*L + *rbc*L dataset of *Campanula* species, the *trn*H-*psb*A marker in *Teucrium* section *Polium*, and selected members of *Scutellaria* section *Scutellaria*. The Jukes–Cantor model was used for the *rbc*L marker in selected members of *Scutellaria* section *Scutellaria*. Node support was assessed using 100 bootstrap replicates, and all sites were included as a gap treatment approach.

## 3. Results

All local endemic Cretan taxa examined are currently maintained under ex situ conservation (eight taxa are first-time reported under ex situ conservation) at the Institute of Plant Breeding and Genetic Resources of the Hellenic Agricultural Organization Dimitra (ELGO-Dimitra) in Thessaloniki, Greece; they all have detailed and fully documented information regarding their origin and identity (location, date of collection, collectors, reference code, taxonomic identification) encoded with IPEN (International Plant Exchange Network) accession numbers. However, full documentation was not always available for the sequences retrieved from the GenBank in comparison with the sequences produced in this study.

### 3.1. PCR and Sequencing

The PCR amplification of the three DNA barcode regions (*rbc*L, *trn*L, *trn*H-*psb*A) was successful in all cases of the 15 Cretan taxa studied herein. Sequencing was also successful for all cases and all three markers, except *Scutellaria sieberi* in *rbc*L.

The *rbc*L region showed the smallest length variation among the studied taxa, ranging from 637 bp in *Phlomis lanata* to 668 bp in *Helichrysum heldreichii*. In contrast, the *trn*L marker presented significant length variation among the examined taxa, spanning from 394 bp in *Ebenus cretica* to 568 bp in *Salix kaptarae*. Lastly, the *trn*H-*psb*A marker exhibited remarkable length divergence among the studied taxa, varying from 168 bp in *Ebenus cretica* to 586 bp in *Sesleria doerfleri*.

### 3.2. Marker-Specific Discriminatory Performance

**Figure 2:**
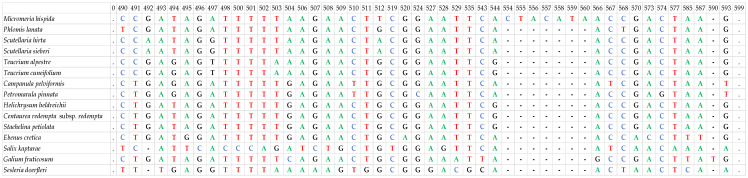
Indicative polymorphisms identified in the *trn*L region among the examined 15 local endemic taxa of Crete (due to the high number of polymorphic sites only the ones in a 100 bp part of the sequence are shown). Each letter/color represents a nucleotide (C: Cytosine, T: Thymine, A: Adenine, G: Guanine), dots represent the existence of the same nucleotide for every taxon and hyphens represent gaps in the sequence.

**Figure 3:**
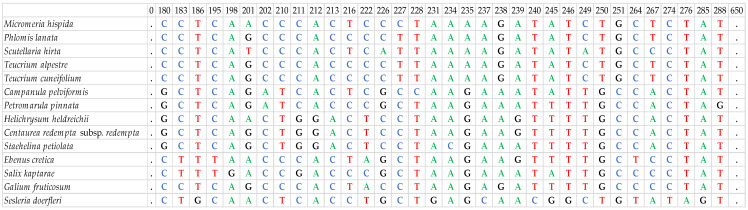
Indicative polymorphisms identified in the *rbc*L region among the examined 15 local endemic taxa of Crete (due to the high number of polymorphic sites only the ones in a 100 bp part of the sequence are shown). Each letter/color represents a nucleotide (C: Cytosine, T: Thymine, A: Adenine, G: Guanine) and dots represent the existence of the same nucleotide for every taxon.

The *rbc*L sequences (650 bp) alignment showed numerous SNPs among the studied taxa, whereas the *trn*L (394–568 bp) alignment exhibited numerous indels and SNPs. Based on these, identification was possible for all studied taxa at the genus level, since the *Teucrium* spp. had no sequence variation at both *rbc*L and *trn*L, while the *Scutellaria* spp. had no sequence variation at *trn*L (Figure 2 and Figure 3).

**Figure 4:**
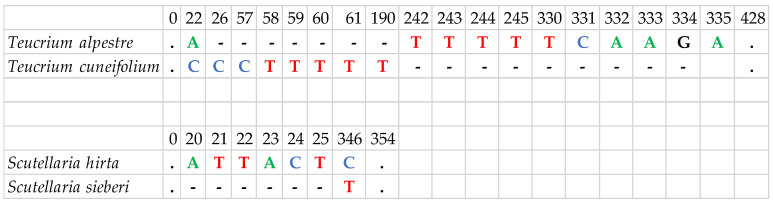
Nucleotide differences identified between species of the genera *Teucrium* and *Scutellaria* for the *trn*H-*psb*A marker. Each letter/color represents a nucleotide (C: Cytosine, T: Thymine, A: Adenine, G: Guanine), dots represent the existence of the same nucleotide for every taxon and hyphens represent gaps in the sequence.

In contrast to the above-mentioned, the *trn*H-*psb*A region (168–586 bp) showed substantial variation in sequence length among species, preventing reliable alignment even at the family level; therefore, phylogenetic analyses were carried out only at the genus level. Furthermore, *trn*H-*psb*A was the only marker capable of distinguishing species within the same genus. Specifically, *Teucrium cuneifolium* and *Teucrium alpestre* differed by one SNP and five indels, while *Scutellaria hirta* and *Scutellaria sieberi* were differentiated in one SNP and one indel (Figure 4). This result highlights the contrasting role of *trn*H-*psb*A compared to the more conserved markers, emphasizing its potential value for species-level identification despite its limitations for broader phylogenetic analyses.

### 3.3. Comparison to the GenBank Sequences

The BLASTn algorithm was used to check the taxonomic identification of the sequences of the studied taxa compared to already deposited GenBank sequences, based on the three DNA regions examined. Regarding the *rbc*L marker, the sequences of *Campanula pelviformis*, *Ebenus cretica* and *Petromarula pinnata* were identified at the species level (accession numbers EU713350.1, OZ197285.1, EU713433.1, respectively). *Scutellaria sieberi* (Lamiaceae) was identified at a 100% query cover and 100% sequence identity as *Sarcopoterium spinosum* (L.) Spach (KY419948.1), *Bencomia sphaerocarpa* Svent. (KY419986.1), *Bencomia exstipulata* Svent. (NC_039924.1), *Sanguisorba menendezii* (Svent.) Nordborg (KY419966.1, synonym of *Dendriopoterium menendezii* Svent.) and *Marcetella moquiniana* (Webb & Berthel.) Svent. (KY420023.1). However, all the above species belong to Rosaceae family and not to Lamiaceae (Table 4). Due to this inconsistency, the sequence was excluded from further analyses. The rest of the studied taxa were correctly identified at the genus level.

For the *trn*L marker, six taxa were correctly identified at the species level, namely *Campanula pelviformis* (MT599795.1), *Ebenus cretica* (AB854525.1 and OZ197285.1), *Petromarula pinnata* (FJ426585.1), *Scutellaria hirta* (EF546927.1), *Scutellaria sieberi* (PQ059201.1) and *Teucrium alpestre* (JN408597.1). The sequence of *Staehelina petiolata* showed identity with 100% query cover and 99.07% sequence identity with two species of Asteraceae family namely *Berardia lanuginosa* (Lam.) Fiori & Paol. (OL702918.1) and *Onopordum acanthium* L. (OR892018.1 and MN919164.1), while the congeneric species *Staehelina uniflosculosa* Sm., AY772368.1 had a 96% query cover and 100% sequence identity (Table 4). The rest of the studied taxa were correctly identified at the genus level.

Regarding the *trn*H-*psb*A marker, three taxa were correctly identified at the species level, namely *Ebenus cretica* (OZ197285.1), *Petromarula pinnata* (MH749227.1) and *Scutellaria sieberi* (PQ059201.1 and PQ059342.1). *Centaurea redempta* subsp. *redempta* had the highest sequence identity (95.76%) with *Carduncellus pinnatus* (Desf.) DC. (HE602494.1; synonym of *Carthamus pinnatus* Desf., i.e., a species belonging to a different genus), while it had 95.08% identity percentage with *Centaurea glastifolia* L. (PQ461097.1). This suggests insufficient divergence of the *trn*H-*psb*A region among closely related genera within Asteraceae (Table 4). *Micromeria hispida* had a 100% query cover and 100% sequence identity with *Thymus linearis* Benth. (LC528389.1) and a 95% query cover and 100% sequence identity with *Micromeria graeca* Benth. ex Rchb. (HQ902830.1), indicating potential phylogenetic proximity or taxonomic complexity within the group. Lastly, *Staehelina petiolata* had 100% query cover and 99.07% sequence identity with multiple species of the genera *Cirsium* and *Carduus*, further supporting the notion that this marker may fail to resolve relationships within certain clades of Asteraceae (Table 4). The rest of the studied taxa were correctly identified at the genus level.

All the sequences obtained for the 15 local endemic taxa of Crete in all markers were submitted to GenBank and obtained respective accession numbers (Table 5). From the comparison of the sequences produced in the present work against the GenBank records, several cases of new genetic data for the studied taxa were revealed, such as species-specific sequences first-time recorded (I), new marker-specific sequences (II), sequences of novel genotypes for DNA markers already represented in GenBank (III), and confirmatory sequences of the same genotype already available in the GenBank (IV) (Table 6). More specifically, for *Centaurea redempta* subsp. *redempta*, *Galium fruticosum*, *Micromeria hispida*, *Salix kaptarae*, and *Teucrium cuneifolium*, new genetic sequences were submitted for the first time, since there was no previous genetic information for any molecular marker available in GenBank. Novel sequences for three additional molecular markers (*rbc*L, *trn*L, *trn*H-*psb*A) were submitted for *Sesleria doerfleri* and *Helichrysum heldreichii*, while sequences for the *rbc*L and *trn*L markers were first-time submitted for *Staehelina petiolata* and for the *rbc*L and *trn*H-*psb*A markers regarding *Scutellaria hirta* and *Teucrium alpestre*. Sequences for the *trn*H-*psb*A marker and for *rbc*L were added for *Campanula pelviformis* and *Phlomis lanata*, respectively (Table 6). Sequences of novel genotypes for the *trn*H-*psb*A marker were submitted for *Staehelina petiolata* and *Scutellaria sieberi* and of *trn*L and *trn*H-*psb*A markers for *Phlomis lanata*; in these cases, differences were found between the sequences of the present study and those already available. Finally, the *rbc*L and *trn*L sequences obtained for *Campanula pelviformis* were congruent with the existing GenBank accessions. Similarly, the *trn*L marker sequences for *Scutellaria hirta*, *Scutellaria sieberi*, and *Teucrium alpestre* showed 100% homology with previously deposited data. For *Ebenus cretica* and *Petromarula pinnata*, all sequenced markers matched the established genotypes currently available in the NCBI database, thereby validating the taxonomic identity of the sampled material (Table 6).

Compared to the already existing genetic data in GenBank, sequences of novel genotypes were added for three species (*Staehelina petiolata*, *Scutellaria sieberi*, *Phlomis lanata*). Specifically, in *S. petiolata* and *S. sieberi*, the additional genotype was recorded in the *trn*H-*psb*A marker, while in *P. lanata,* the additional genotypes were detected in the *trn*H-*psb*A and *trn*L markers.

Compared to the existing genetic data, two insertions in *S. sieberi*, three deletions in *Staehelina petiolata*, *Scutellaria sieberi* and *Phlomis lanata*, and eleven base substitutions (five in *S. sieberi* and six in *P. lanata*) were recorded in total. Among these, only one deletion was found in the *trn*L marker, while all other polymorphisms were observed in the *trn*H-*psb*A marker (Table 7).

### 3.4. Phylogenetic Analyses

**Figure 5:**
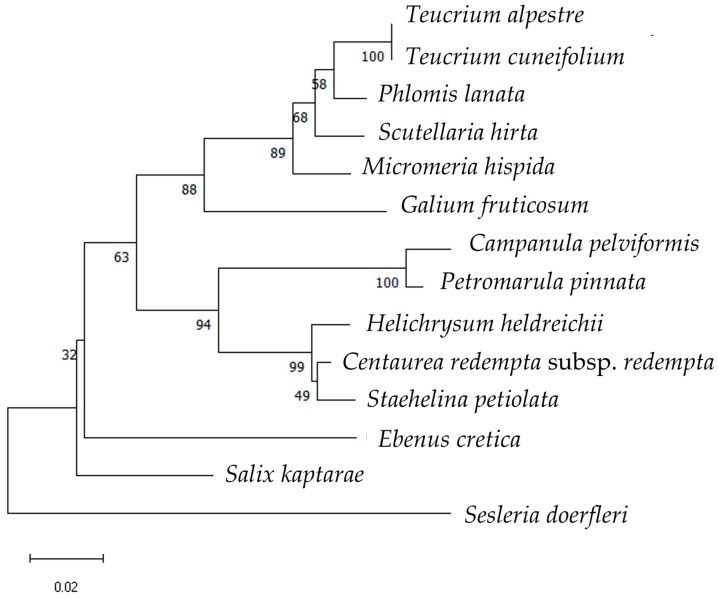
Phylogenetic tree using the *rbc*L sequences (all produced in this investigation) of 14 local endemic taxa of Crete. The phylogeny was inferred using the Maximum Likelihood method and Kimura-2–Gamma Distributed (K2 + G) model of nucleotide substitution, and the percentage of replicate trees in which the associated taxa clustered together (100 replicates) is shown next to the branches.

Comparative phylogenetic trees were constructed with the sequences of the investigated 15 local endemic plants of Crete to illustrate their distinctiveness and evolutionary relationships. In *rbc*L (650 bp; Figure 5), taxa belonging to the same families were grouped together (members of the Lamiaceae, Asteraceae and Campanulaceae families) Every taxon was discerned, except for the members of the same genus, i.e., *T. cuneifolium* and *T. alpestre*. The bootstrap support of the *rbc*L tree showed moderate (e.g., 89, 88) to strong values (e.g., 100, 99, 94) for several nodes, suggesting these relationships are reliable, while lower support values (e.g., 68, 63, 58, 49, 32) suggest uncertainty in those relationships.

**Figure 6:**
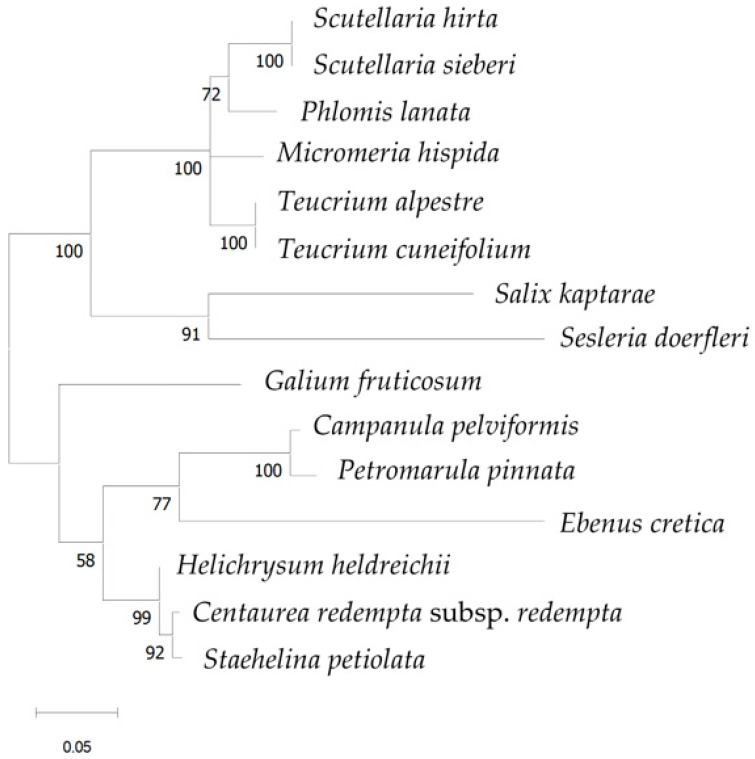
Phylogenetic tree using the *trn*L sequences (all produced in this investigation) of the 15 local endemic taxa of Crete. The phylogeny was inferred using the Maximum Likelihood method and Hasegawa–Kishino–Yano–Gamma-Distributed with Invariant Sites (HKY + G + I) model of nucleotide substitution, and the percentage of replicate trees in which the associated taxa clustered together (100 replicates) is shown next to the branches.

In *trn*L (394–468 bp; Figure 6), a clustering of closely-related taxa (e.g., taxa belonging to the same family) could also be noticed mainly in moderate (e.g., 72, 77) to high (e.g., 100, 91, 99, 92) bootstrap support, but taxa belonging to the same genera (*Scutellaria hirta*, *S. sieberi* and *Teucrium cuneifolium*, *T. alpestre*) could not be distinguished.

**Figure 7:**
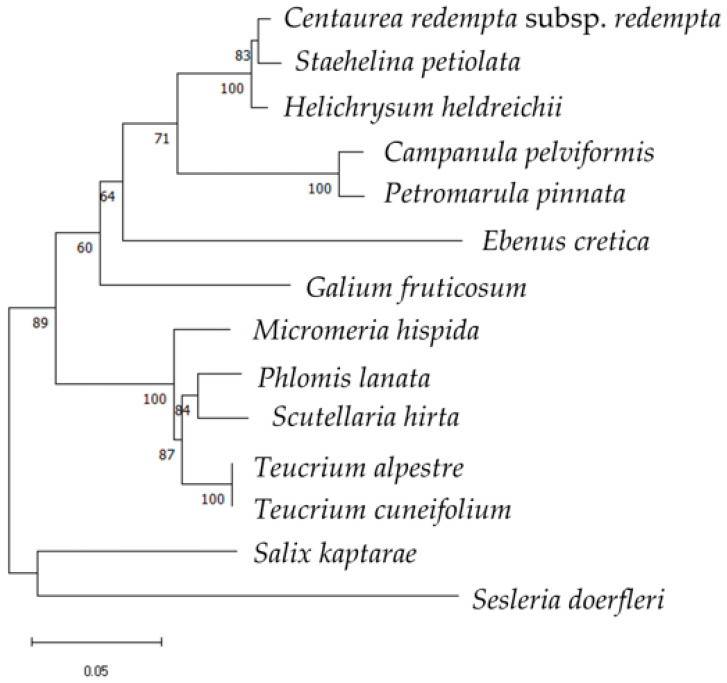
Phylogenetic tree using the *rbc*L and *trn*L sequences (all produced in this investigation) of 14 local endemic taxa of Crete. The phylogeny was inferred using the Maximum Likelihood method and Hasegawa–Kishino–Yano–Gamma-Distributed with Invariant Sites (HKY + G + I) model of nucleotide substitution, and the percentage of replicate trees in which the associated taxa clustered together (100 replicates) is shown next to the branches.

The phylogenetic tree constructed from the combined *rbc*L and *trn*L sequences (1044–1218 bp; Figure 7) provided a result similar to the *trn*L marker (Figure 6), with an average of lower branch support.

The phylogenetic tree for the *trn*H-*psb*A marker (168–586 bp) could not be constructed due to large differences in the length of the sequences among the examined taxa.

**Figure 8:**
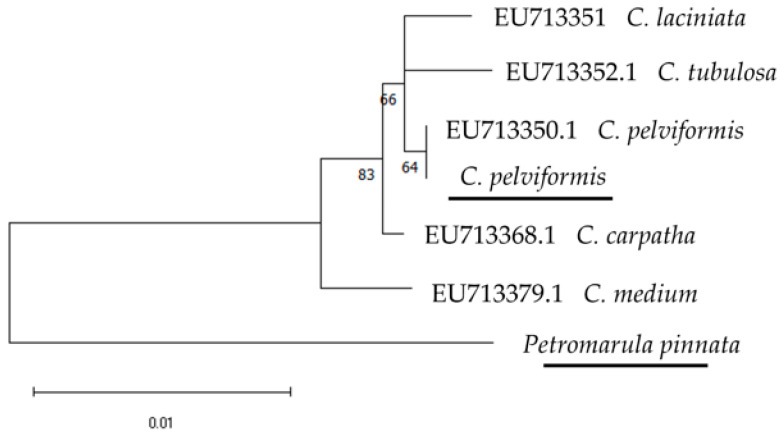
Phylogenetic tree using the *rbc*L and *trn*L markers of selected members belonging to the section *Quinqueloculares* of the genus *Campanula*, with *Petromarula pinnata* (also Campanulaceae) as an outgroup (underlined: produced in this investigation). The phylogeny was inferred using the Maximum Likelihood method and Hasegawa–Kishino–Yano with Invariant Sites (HKY + I) model of nucleotide substitution, and the percentage of replicate trees in which the associated taxa clustered together (100 replicates) is shown next to the branches.

Based on the sequences acquired, targeted phylogenetic analyses were carried out for some of the taxa studied herein, exhibiting difficulties in their morphological identification, as they showed similarities to other species belonging to the same section. For this purpose, sequences of 44 additional taxa of the studied sections were added in these analyses. In this way, a phylogenetic tree was constructed based on the *rbc*L and *trn*L sequences (1144–1153 bp) for *Campanula pelviformis* and four additional taxa belonging to the section *Quinqueloculares*, with *Petromarula pinnata* as an outgroup (sequence of the present study) (Figure 8). On this tree, the two sequences of *C. pelviformis* (the one retrieved from the GenBank, and the one acquired in the present study) were similar, while all five taxa of the section *Quinqueloculares* were discriminated, with moderate bootstrap values (*C. medium* L. *C. carpatha* Halácsy, *C. laciniata* L., *C. tubulosa* Lam., Figure 8).

**Figure 9:**
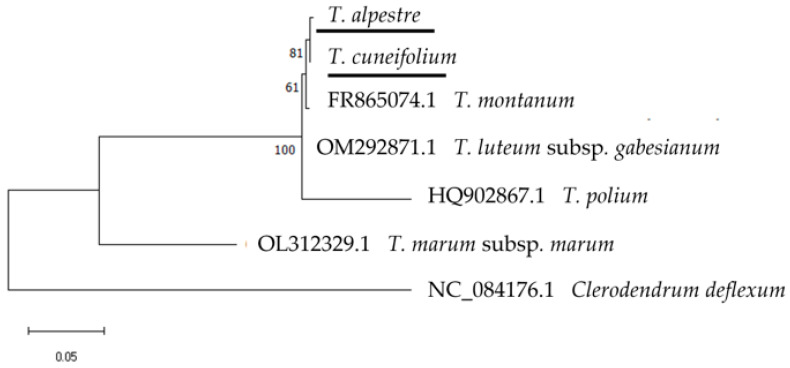
Phylogenetic tree using the *trn*H-*psb*A marker of *Teucrium* section *Polium* members including the herein studied Cretan endemic *T. alpestre* and *T. cuneifolium* (underlined), with *Clerodendrum deflexum* Wall. (also, Lamiaceae) as an outgroup. The phylogeny was inferred using the Maximum Likelihood method and Hasegawa–Kishino–Yano with Invariant Sites (HKY + I) model of nucleotide substitution, and the percentage of replicate trees in which the associated taxa clustered together (100 replicates) is shown next to the branches.

Furthermore, phylogenetic trees using the sequences of *trn*L and *rbc*L markers (either individually or in combination) were constructed for taxa belonging to the section *Polium* of the genus *Teucrium*. In the *trn*L marker (447–449 bp), three sect. *Polium* members (*T. alpestre*, *T. cuneifolium*, *T. polium* L.) were clustered together with no apparent differences between them, while the remaining three taxa were discerned (Supplementary Materials, Figure S1). In the *rbc*L marker (516 bp), five of the six taxa namely *T. alpestre*, *T. cuneifolium*, *T. luteum* (Mill.) Degen subsp. *gabesianum* (S.Puech) Greuter, *T. montanum* L., and *T. polium* were grouped together with identical sequences, while only *T. marum* L. subsp. *marum* was differentiated (Supplementary Materials, Figure S2). In contrast, all the taxa were discerned in the *trn*H-*psb*A marker (385–415 bp) with moderate to strong bootstrap values (Figure 9). The combination of the *trn*L and *rbc*L markers (963–965 bp) could not discriminate the taxa due to the absolute similarity of the sequences of *T. alpestre*, *T. cuneifolium*, and *T. polium* in these two markers, thus providing a result like the one obtained for the *trn*L marker (Supplementary Materials, Figure S3).

**Figure 10:**
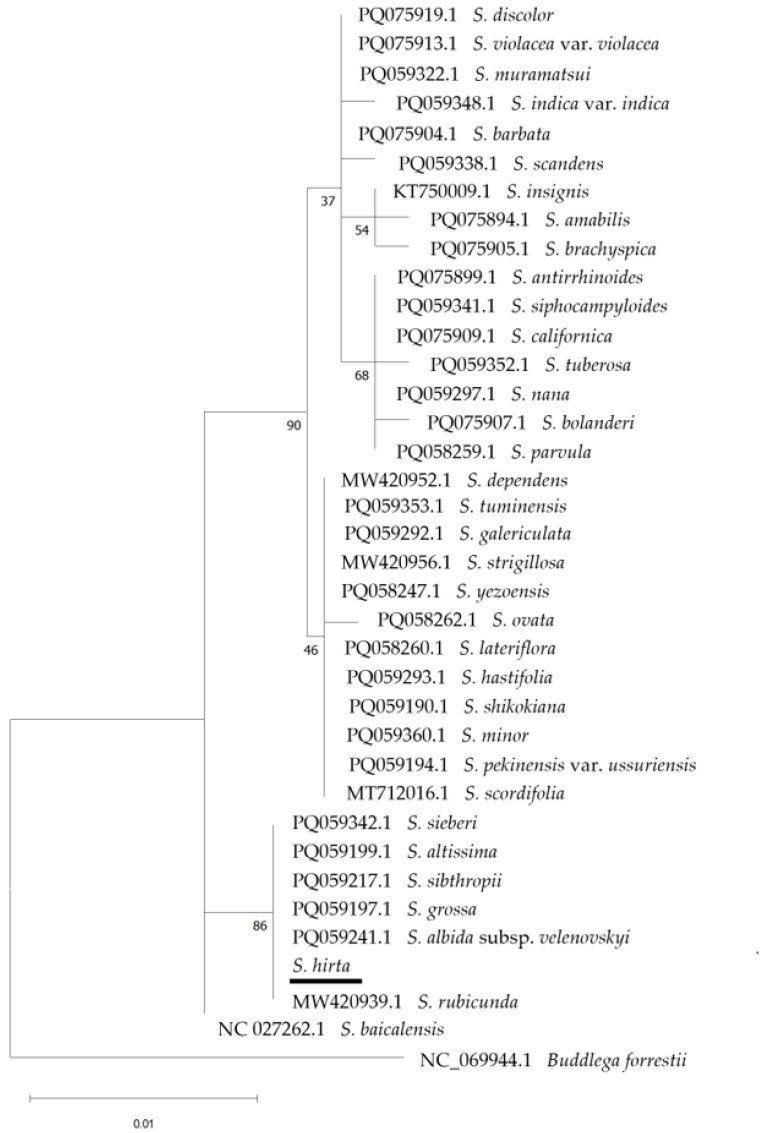
Phylogenetic tree using the *rbc*L marker with selected *Scutellaria* section *Scutellaria* members including the herein studied Cretan endemic *S. hirta* and *S. sieberi* (underlined), with *Buddleja forrestii* Diels (Scrophulariaceae) as an outgroup. The phylogeny was inferred using the Maximum Likelihood method and the Jukes–Cantor model of nucleotide substitution, and the percentage of replicate trees in which the associated taxa clustered together (100 replicates) is shown next to the branches.

**Figure 11:**
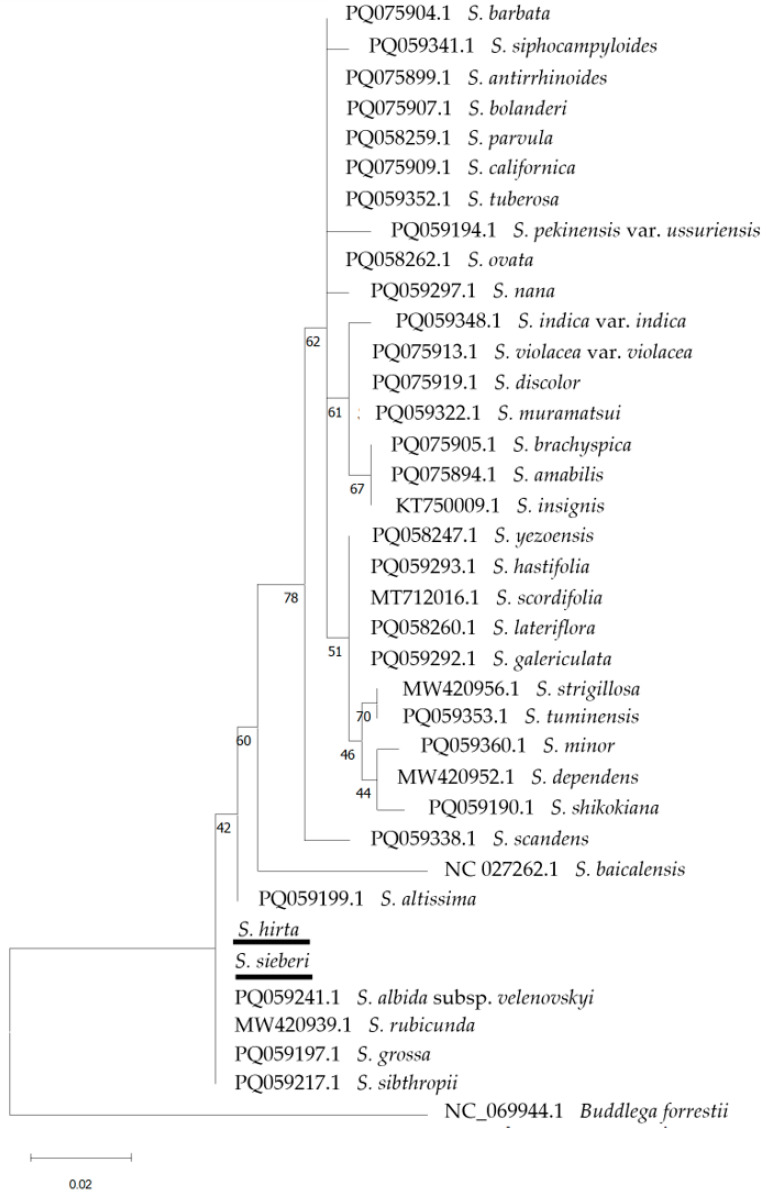
Phylogenetic tree using the *trn*H-*psb*A marker with selected *Scutellaria* section *Scutellaria* members including the herein studied Cretan endemic *S. hirta* and *S. sieberi* (underlined), with *Buddleja forestii* as an outgroup. The phylogeny was inferred using the Maximum Likelihood method and Hasegawa–Kishino–Yano with Invariant Sites (HKY + I) model of nucleotide substitution, and the percentage of replicate trees in which the associated taxa clustered together (100 replicates) is shown next to the branches.

In total, 37 taxa belonging to section *Scutellaria* of the genus *Scutellaria* (including the Cretan endemics *S. hirta* and *S. sieberi*) were used to create phylogenetic trees for the examined markers individually, as well as for the combination of *trn*L and *rbc*L markers. In the phylogenetic tree resulting from the *trn*L marker (457–477 bp), not all *Scutellaria* taxa were discerned, e.g., *S. brachyspica* Nakai & H.Hara, *S. indica* L. var. *indica*, and *S. barbata* D.Don clustering together (Supplementary Materials, Figure S4). In contrast, in the *rbc*L (660 bp; Figure 10) and the *trn*H-*psb*A (197–225 bp; Figure 11) markers, several clusters were formed, but some taxa were not discerned such as *S. parvula* Minchx., *S. siphocampyloides* Vatke, *S. nana* A.Gray, *S. brachyspica*, and *S. insignis* Nakai. The combination of the *trn*L and *rbc*L markers (1117–1137 bp) showed a similar result (Supplementary Materials, Figure S5). In these trees (Figure 10 and Figure 11), while a few major clades are well supported, many internal nodes show low to moderate bootstrap values. This suggests that relationships among several *Scutellaria* species using these markers remain unresolved and should be interpreted with caution.

## 4. Discussion

The International Barcode of Life (iBOL) is an international research alliance involving national institutions and research consortia committed to study biodiversity by developing a globally accessible DNA-based system (short DNA sequences—DNA barcodes) for the genetic identification of all multicellular organisms [46,47]. The development of DNA barcode reference libraries is essential and critical to document and assess biodiversity [48,49], especially for range-restricted taxa with significant ecological importance as well as social and economic benefits, which are one way or another under threat [50]. In this context, the present work contributes to the enrichment of the genetic information database GenBank (NCBI) by adding sequences of three DNA barcodes (*trn*L, *rbc*L, and *trn*H-*psb*A) for 15 plant taxa that are locally endemic to the island of Crete, Greece (single-island endemics with restricted ranges in some parts of the island). The contribution to the enrichment of the Genbank is multifold: (i) submission of sequences recorded for the first time for five taxa (*Centaurea redempta* subsp. *redempta*, *Galium fruticosum*, *Micromeria hispida*, *Salix kaptarae*, *Teucrium cuneifolium*); (ii) submission of sequences for additional molecular markers for seven taxa (*Campanula pelviformis*, *Helichrysum heldreichii*, *Phlomis lanata*, *Sesleria doerfleri*, *Staehelina petiolata*, *Scutellaria hirta*, *Teucrium alpestre*); (iii) submission of sequences of novel genotypes for three taxa (*Phlomis lanata*, *Staehelina petiolata*, *Scutellaria sieberi*); and (iv) submission of confirmatory sequences of extant genotypes for six taxa (*C. pelviformis*, *Ebenus cretica*, *Petromarula pinnata*, *S. hirta*, *S. sieberi*, *T. alpestre*). All the above categories of genetic information are important and necessary for biodiversity conservation and sustainable management, especially when these are focused on local endemic taxa with insular range (single-island endemics). This kind of information may facilitate various applications such as the identification, discrimination, classification and phylogenetic analyses of closely related taxa (e.g., taxa belonging to the same genus or members of closely related genera), the clarification of their evolutionary relationships and origin, and the study of intrageneric polymorphisms, e.g., [15,51,52,53,54,55]. In addition, such information also allows the authentication of plant-derived products [56,57,58], thus contributing to the sustainable utilization of plants with conservation concern; this is especially important in cases of local endemics and/or threatened species like those studied herein, which are also associated with actual or potential economic value in industrial sectors [31,32,33]. In this way, the generated genetic information is made accessible through specialized repositories (NCBI) to the global scientific community, thus facilitating further basic and applied research.

BLASTn searches were conducted to check for taxonomic identification against GenBank sequences; however, in some cases discordant results emerged, where high-similarity matches failed to correspond with anticipated conspecific or congeneric taxa. Such cases were *Micromeria hispida* sharing 100% identity with *Thymus linearis* at the *trn*H-*psb*A marker and *Centaurea redempta* subsp. *redempta* and *Staehelina petiolata* matching other Asteraceae genera better than the congeneric records at the *trn*L and *trn*H-*psb*A markers. Such discrepancies may be attributed to the limited discriminatory power of the marker and/or to the presence of conserved sequences across different genera/species of the same family. However, the discrepancy of *Scutellaria sieberi* matching Rosaceae taxa may be attributed to possible database misannotation or mistakes in plant specimen identification. Lastly, incomplete lineage sorting and taxonomic complexity of certain taxa may not be excluded. Using plastid-only barcoding regions in the present work did not allow for species-level identification in all cases, even though it is supposed that the use of three regions may yield close to optimal discriminatory ability [59]. This result may reflect incomplete lineage sorting, insufficient sequence divergence within the marker, or limited representation of closely related taxa in the database. Thus, the findings of the present study corroborate numerous other studies showing that the use of plastid-only DNA barcoding regions has certain limitations, and it is insufficient for closely related taxa. Furthermore, the consequences of sparse intraspecific sampling or database incompleteness or misannotations can severely compromise the reliability of BLAST-based identification [60]. The incorporation of nuclear markers’ sequences and/or plastome/genome-scale data with NGS approaches will enhance phylogenetic accuracy and improve species discrimination.

Comparative analyses within selected taxa provided a preliminary assessment of chloroplast marker performance and demonstrated that the *trn*L and *rbc*L regions were relatively conserved, providing limited resolution at the species level, either each one alone or in combination, e.g., members of the section *Polium* of the genus *Teucrium*. In contrast, significant intraspecific diversity was observed in the *trn*H-*psb*A region, allowing in most cases discrimination between closely related taxa. This assortment can be attributed to the nature of *trn*H-*psb*A, which corresponds to a non-coding region, in which mutations tend to accumulate at a faster rate due to less stringent DNA repair mechanisms [61]. The presence of length polymorphisms may be attributed to the presence of pseudogenes, elevated insertion–deletion rates, and repeated regions [62]. In addition, length polymorphism in the *trn*H-*psb*A region has been attributed to the inclusion of copies of *rps*19 and associated pseudogenes located between the *trn*H and *psb*A genes, leading to considerable length variation and further complicating interspecific and inter-familial alignments [63].

These findings are consistent with previous studies examining the performance of chloroplast DNA barcodes. For instance, research on native Lamiaceae plants of Chios Island and the adjacent Cesme–Karaburun Peninsula have shown the lowest levels of *rbc*L variability among the studied Lamiaceae taxa and significantly high *trn*H-*psb*A variability, due to the presence of indels [64]. Similarly, another study examining 14 vascular endemic plants from Trinidad has reported that the slow evolutionary rate of *rbc*L may limit its ability to accurately identify species at the genus level [65]. Concerning the *trn*L region, the findings of the present work are consistent with previous studies on DNA barcoding of plant species of the Lamiaceae family [66,67,68] and of flowering plants in different families [38,69] reporting lower *trn*L variability compared to *trn*H-*psb*A. Consequently, it has been argued that the *trn*L intron may not be well suited for identifying closely related species, since it is relatively conserved at this level [70].

Overall, the observed differences in variability among DNA barcoding regions highlight that marker performance is rather taxon-dependent [71]. Consequently, research results derived from one group of endemic plant species cannot be universally applied to others, as species-specific ecological pressures and distinct evolutionary histories can differentially influence patterns of DNA sequence evolution [72]. Future work on endemic plant groups should build upon this reference dataset by incorporating additional loci, particularly nuclear markers or multi-locus plastome/genome-scale approaches to improve species discrimination and provide a more comprehensive understanding of genetic relationships among Cretan endemic taxa.

## 5. Conclusions

The present investigation contributes considerably to the enrichment of the GenBank with new genetic information regarding 15 local endemic plant taxa of Crete, originating from an important global endemism center and biodiversity hotspot. In the present study, the *trn*H-*psb*A region showed the highest species-level discriminatory power, while the *trn*L and *rbc*L markers were effective at the genus level discrimination. Their combination did not provide clear distinction at the species level in all cases. Overall, the phylogenetic information and the effectiveness of DNA barcoding seemed to vary among the DNA regions used, most probably due to the high diversity of the plant taxa in concern (15 taxa of 13 genera in 7 families). Undoubtedly, future studies should prioritize the inclusion of nuclear markers and/or multi-locus genomic markers towards effective species discrimination.

## Figures and Tables

**Table 4:** Discordant matches between the query and subject sequences found by BLASTn searches.

Cretan Endemic Taxon	*rbc*L	*trn*L	*trn*H-*psb*A
*Scutellaria sieberi* (Lamiaceae)	Rosaceae taxa	-	-
*Staehelina petiolata* (Asteraceae)	-	Asteraceae taxa	Taxa of the genera *Cirsium* and *Carduus* (Asteraceae)
*Centaurea redempta* subsp. *redempta* (Asteraceae)	-	-	*Carduncellus pinnatus* (Asteraceae)
*Micromeria hispida* (Lamiaceae)	-	-	*Thymus linearis* (Lamiaceae)

**Table 5:** Accession numbers in GenBank (NCBI) for the deposited sequences of the 15 local endemic taxa of Crete (alphabetically) regarding three DNA barcode markers (*rbc*L, *trn*L, and *trn*H-*psb*A).

Taxon	*rbc*L	*trn*L	*trn*H-*psb*A
*Campanula pelviformis*	PV605675	PV605661	PV605688
*Centaurea redempta* subsp. *redempta*	PV605676	PV605662	PV605689
*Ebenus cretica*	PV605677	PV605663	-
*Galium fruticosum*	PV605678	PV605664	PV605690
*Micromeria hispida*	PV605679	PV605665	-
*Petromarula pinnata*	PV605680	PV605666	PV605691
*Phlomis lanata*	PV605681	PV605667	PV605692
*Salix kaptarae*	PV605682	PV605668	PV605693
*Scutellaria hirta*	PV605683	PV605669	PV605694
*Scutellaria sieberi*	-	PV605670	PV605695
*Sesleria doerfleri*	PV605684	PV605671	PV605696
*Staehelina petiolata*	PV605685	PV605672	PV605697
*Teucrium alpestre*	PV605686	PV605673	PV605698
*Teucrium cuneifolium*	PV605687	PV605674	PV605699

**Table 6:** Characterization of the genetic data on three DNA barcode markers (*rbc*L, *trn*L, and *trn*H-*psb*A) submitted in GenBank for the 15 local endemic taxa of Crete: (I) sequences recorded for the first time; (II) novel sequences of new molecular markers; (III) sequences of novel genotypes for DNA markers already represented in the GenBank; (IV) confirmatory sequences of the same genotype already available in the GenBank.

Taxon	*rbc*L	*trn*L	*trn*H-*psb*A
*Campanula pelviformis*	IV	IV	II
*Centaurea redempta* subsp. *redempta*	I	I	I
*Ebenus cretica*	IV	IV	IV
*Galium fruticosum*	I	I	I
*Helichrysum heldreichii*	II	II	II
*Micromeria hispida*	I	I	I
*Petromarula pinnata*	IV	IV	IV
*Phlomis lanata*	II	III	III
*Salix kaptarae*	I	I	I
*Scutellaria hirta*	II	IV	II
*Scutellaria sieberi*	-	IV	III
*Sesleria doerfleri*	II	II	II
*Staehelina petiolata*	II	II	III
*Teucrium alpestre*	II	IV	II
*Teucrium cuneifolium*	I	I	I

**Table 7:** Differences in genotypes of the three local endemic taxa of Crete in *rbc*L, *trn*L, and *trn*H-*psb*A compared to already available sequences in GenBank. References: [42,43,44,45].

Taxon	DNA Marker	Sequence ID	Polymorphism Position	Polymorphism Type	Difference	Reference
*Staehelina petiolata*	*trn*H-*psb*A	EU571456.1	257	Deletion	C	[42]
*Scutellaria sieberi*	*trn*H-*psb*A	PQ059342.1	20–25	Insertion	ATTACT	[43]
*Scutellaria sieberi*	*trn*H-*psb*A	PQ059201.1	20–25	Insertion	ATTACT	[43]
*Scutellaria sieberi*	*trn*H-*psb*A	PQ059202.1	20	Substitution	A instead of T	[43]
*Scutellaria sieberi*	*trn*H-*psb*A	PQ059202.1	24–33	Deletion	AATTCATTTT	[43]
*Scutellaria sieberi*	*trn*H-*psb*A	PQ059202.1	34	Substitution	C instead of T	[43]
*Scutellaria sieberi*	*trn*H-*psb*A	PQ059202.1	39	Substitution	A instead of T	[43]
*Scutellaria sieberi*	*trn*H-*psb*A	PQ059202.1	44	Substitution	C instead of A	[43]
*Scutellaria sieberi*	*trn*H-*psb*A	PQ059202.1	262	Substitution	G instead of T	[43]
*Phlomis lanata*	*trn*L	GU993211.1	94–96	Deletion	TTT	[44]
*Phlomis lanata*	*trn*H-*psb*A	KP835752.1	48	Substitution	T instead of G	[45]
*Phlomis lanata*	*trn*H-*psb*A	KP835752.1	66	Substitution	T instead of G	[45]
*Phlomis lanata*	*trn*H-*psb*A	KP835751.1	48	Substitution	T instead of G	[45]
*Phlomis lanata*	*trn*H-*psb*A	KP835750.1	48	Substitution	T instead of G	[45]
*Phlomis lanata*	*trn*H-*psb*A	KP835749.1	48	Substitution	T instead of G	[45]
*Phlomis lanata*	*trn*H-*psb*A	KP835748.1	48	Substitution	T instead of G	[45]

## Data Availability

The original data presented in this study are included in the article and its Supplementary Materials, and the genetic sequences have been deposited to GenBank (NCBI). Further inquiries can be directed at the corresponding authors.

## Supplementary Materials

The following supporting information can be downloaded at https://www.mdpi.com/article/10.3390/cimb48050500/s1.
