# Supplementary material for: DNA Barcoding and Comparative Chloroplast Marker Performance in Endemic Plants of Crete (Greece)
Source: Curr Issues Mol Biol. 2026 May 13;48(5):500. doi: 10.3390/cimb48050500 (PMC13204560; doi:10.3390/cimb48050500)
Supplement: Supplementary file 1 [file cimb-48-00500-s001.zip › cimb-4259240-supplementary.pdf]

Article

DNA Barcoding and Comparative Chloroplast Marker Performance in Endemic Plants of Crete (Greece)

Dimitra Ioannidou[1], Ioulietta Samartza[2], Georgios Tsoktouridis[2], Andreas D. Drouzas[1,*], Nikos Krigas[2,3,4,*]

[1] Laboratory of Systematic Botany and Phytogeography, Department of Botany, School of Biology, Aristotle University of Thessaloniki, 54124 Thessaloniki, Greece; drouzas@bio.auth.gr (A.D.D.); demy.ioann@gmail.com (D.I.)

[2] Institute of Plant Breeding and Genetic Resources, Hellenic Agricultural Organization Demeter (ELGO-Dimitra), Thermi, 57001 Thessaloniki, Greece; isamartza@elgo.gr (I.S.); gtsok@elgo.gr (G.T.)

[3] Institute of Olive Tree, Subtropical Crops and Viticulture, Hellenic Agricultural Organization Demeter (ELGO-Dimitra), 71307 Heraklion; nkrigas@elgo.gr (N.K.)

[4] Department of Agriculture, School of Agricultural Sciences, Hellenic Mediterranean University, 71410 Heraklion, Greece; nkrigas@hmu.gr (N.K.)

* **Correspondence: drouzas@bio.auth.gr (A.D.D.); nkrigas@elgo.gr (N.K.)**

Supplementary materials

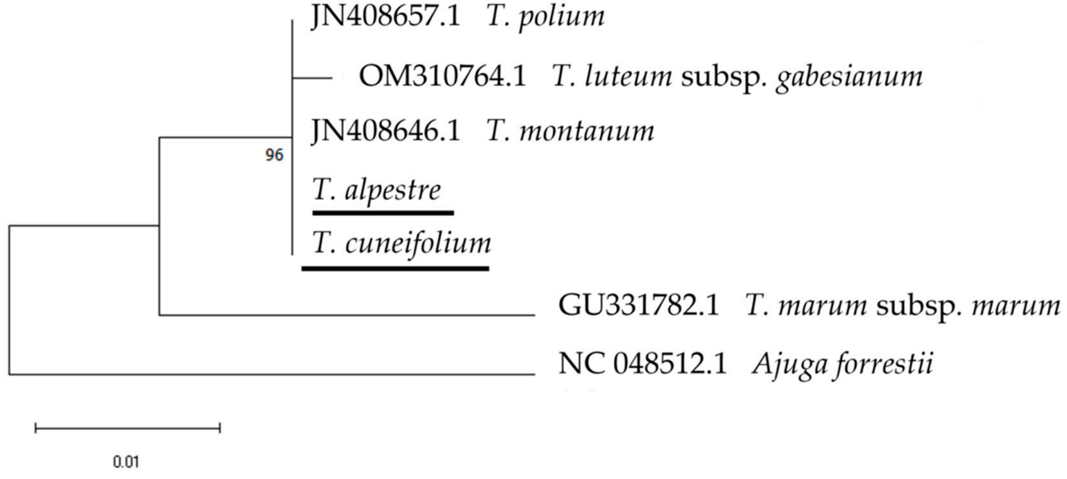

Figure S1. Phylogenetic tree using the *trn*L marker of *Teucrium* Section *Polium* members including the Cretan endemics *T. alpestre* and *T. cuneifolium* (underlined), with *Ajuga forrestii* Diels (also Lamiaceae) as an outgroup. The phylogeny was inferred using Maximum Likelihood method and Hasegawa-Kishino-Yano (HKY) model of nucleotide substitution and the percentage of replicate trees in which the associated taxa clustered together (100 replicates) is shown next to the branches.

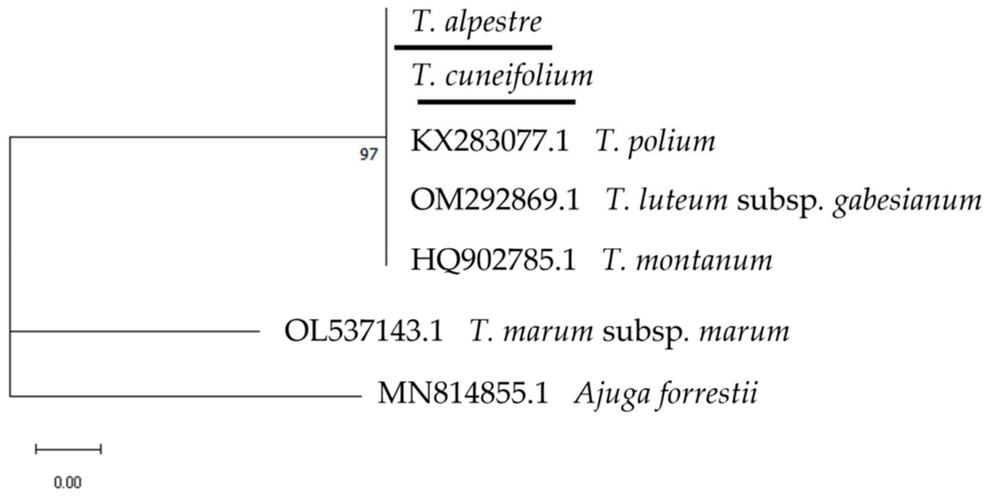

Figure S2. Phylogenetic tree using the *rbc*L marker of selected *Teucrium* section *Polium* members including the Cretan endemics *T. alpestre* and *T. cuneifolium* (underlined), with *Ajuga forrestii* (also Lamiaceae) as an outgroup. The phylogeny was inferred using Maximum Likelihood method and Jukes-Cantor (JC) model of nucleotide substitution and the percentage of replicate trees in which the associated taxa clustered together (100 replicates) is shown next to the branches.

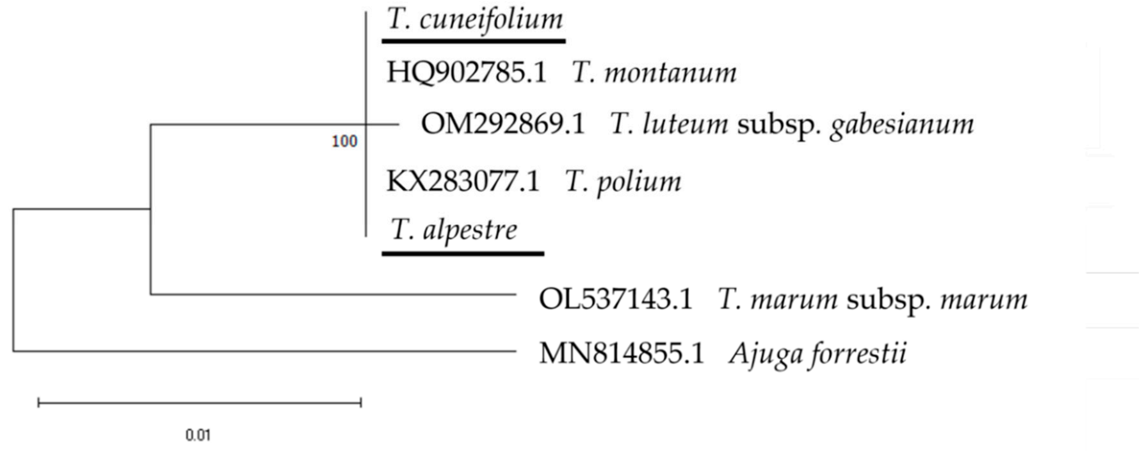

Figure S3. Phylogenetic tree using the *trn*L and *rbc*L markers of selected *Teucrium* Section *Polium* members including the Cretan endemics *T. alpestre* and *T. cuneifolium* (underlined), with *Ajuga forrestii* (also Lamiaceae) as an outgroup. The phylogeny was inferred using Maximum Likelihood method and Hasegawa-Kishino-Yano (HKY) model of nucleotide substitution and the percentage of replicate trees in which the associated taxa clustered together (100 replicates) is shown next to the branches

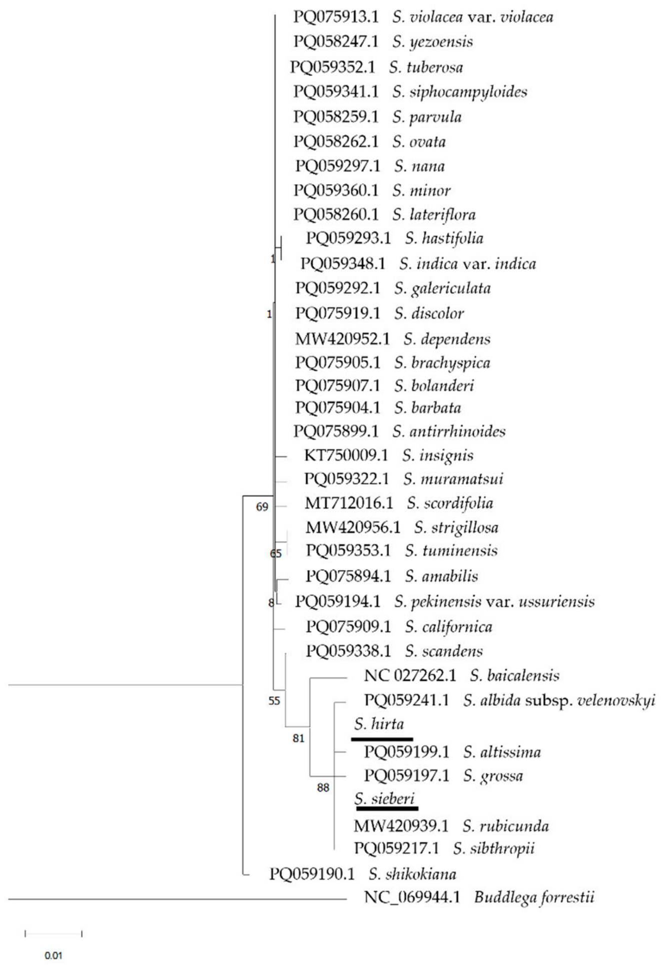

Figure S4. Phylogenetic tree using the *trn*L marker with selected *Scutellaria* Section *Scutellaria* members including the Cretan endemics *S. hirta* and *S. sieberi* (underlined), with *Buddleja forrestii* Diels (Scrophulariaceae) as an outgroup. The phylogeny was inferred using Maximum Likelihood method and Hasegawa-Kishino-Yano - Gamma Distributed with Invariant Sites (HKY+G+I) model of nucleotide substitution and the percentage of replicate trees in which the associated taxa clustered together (100 replicates) is shown next to the branches.

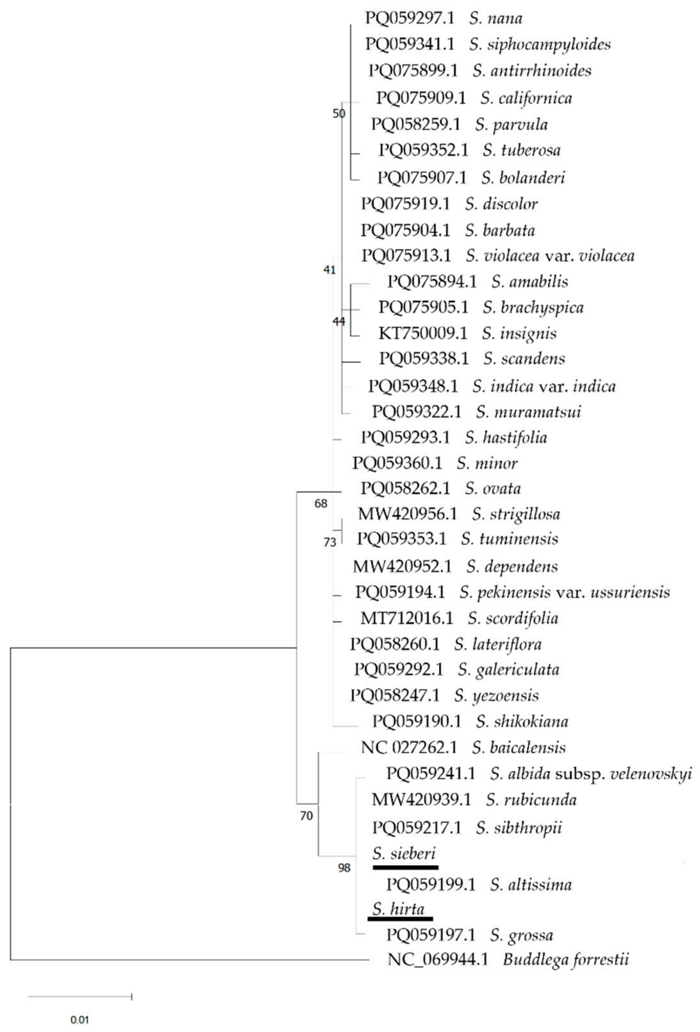

Figure S5. Phylogenetic tree using the *trn*L and *rbc*L markers with selected *Scutellaria* Section *Scutellaria* members including the Cretan endemics *S. hirta* and *S. sieberi*, with *Buddleja forrestii* (Scrophulariaceae) as an outgroup. The phylogeny was inferred using Maximum Likelihood method and Hasegawa-Kishino-Yano - Gamma Distributed with Invariant Sites (HKY+G+I) model of nucleotide substitution and the percentage of replicate trees in which the associated taxa clustered together (100 replicates) is shown next to the branches.
